# Evaluation of standard use of intravenous infusion in 25 general hospitals in Shanxi province based on factor analysis and cluster analysis

**DOI:** 10.3389/fphar.2025.1708461

**Published:** 2025-11-13

**Authors:** Lin Chen, Song Wang, Jinju Duan, Rong Yu, Ruigang Hou, Donghong Yin

**Affiliations:** 1 School of Pharmacy, Shanxi Medical University, Taiyuan, Shanxi, China; 2 Department of Pharmacy, Second Hospital of Shanxi Medical University, Taiyuan, Shanxi, China; 3 Department of Pharmacy, Shanxi Cardiovascular Hospital, Taiyuan, Shanxi, China; 4 Shanxi Pharmacy Administration and Quality Control Center, Taiyuan, Shanxi, China

**Keywords:** intravenous infusion, inpatients, general hospitals, principal component analysis, factor analysis, cluster analysis

## Abstract

**Introduction:**

Intravenous (IV) infusion is overused in Chinese hospitals, and a tool to appraise its appropriateness quantitatively and qualitatively is lacking. This study aimed to develop a multidimensional evaluation framework for assessing the appropriateness of IV infusion use in general hospitals using factor and cluster analyses.

**Methods:**

We conducted a multicentre retrospective study using data from 25 hospitals and a total of 2,064 cases were analyzed. Stratified proportional random sampling was used to select medical records, and a dedicated intravenous infusion survey form was completed for each patient. Factor analysis and cluster analysis were employed to comprehensively assess the ranking and classification of standardized intravenous infusion use among these hospitals. The Kruskal-Wallis test was used to compare disparities in performance characteristics across clusters. Nine IV infusion-related indicators were selected based on national quality control guidelines. Principal component factor analysis was used to extract common factors, and K-means cluster analysis was applied to categorize hospitals into performance tiers. Statistical comparisons were performed to validate inter-group differences.

**Results:**

The number of prescription items was classified according to ATC codes, with the top three being anti-infectives (J01), nutritional preparations (V06), and vitamins (A11). Two factors explained 81.9% of variance: “intensity” (high loadings on DOT, LOT, volume, bottles) and “penetration” (utilization and bed-day coverage). K-means clustering classified the 25 hospitals into four categories. “Excellent” (24.00%), “good” (20.00%), “Middle” (2.00%), and “Inferior” (48.00%). Inter-group comparisons showed no statistically significant difference in the proportion of intravenous infusion orders (P = 0.131), while the other eight indicators differed significantly (P < 0.01). These results objectively reflect the distinct characteristics of intravenous infusion practices among hospitals in Shanxi Province.

**Discussion:**

The factor analysis incorporating nine evaluation indicators can serve as a method to assess the standardization of intravenous infusion in hospitals. The cluster analysis reveals a discernible pattern in the combination of intravenous-infusion -related indicators across hospitals. This study presents a practical and scalable tool for evaluating IV infusion appropriateness in hospitals. The proposed framework supports targeted quality improvement interventions and aligns with national goals for rational drug use. Future efforts should expand the scope of evaluation and link infusion patterns to clinical outcomes.

## 1 Introduction

Intravenous (IV) infusion, owing to its advantages such as rapid onset of action and high bioavailability, has become one of the most commonly used drug administration routes for hospitalized patients. Particularly in critical care, IV infusion can rapidly achieve effective plasma drug concentrations, playing an irreplaceable role in saving lives (Alexander et al., 2021; Haas, 2017). However, intravenous drug administration is an invasive procedure that increases the risks of phlebitis, drug extravasation, infection, and adverse drug reactions (ADRs) (Valderas et al., 2018; Brkić, 2019). The irrational and excessive use of IV infusion not only fails to improve treatment outcomes for patients but also tends to induce adverse reactions, posing more safety hazards (Kellum et al., 2015; Liu et al., 2025). According to the 2024 Annual Report of the National Adverse Drug Reaction Monitoring Center, China National Center for ADR Monitoring received a total of 2.597 million “Adverse Drug Reaction/Event Report Forms,” with ADRs induced by injection administration accounting for 56.3% of all reports, of which intravenous injection accounted for 91.1% (NMPA, 2024). Moreover, excessive infusion not only prolongs hospital stays and increases patients’ financial burdens but also contradicts the World Health Organization’s principle of “oral administration preferred over intramuscular injection, and intramuscular injection preferred over intravenous infusion”.

The Chinese government is highly concerned about the excessive use of IV infusion among hospitalized patients. Since 2021, reducing the utilization rate of IV infusion has been set as one of the top ten national medical quality and safety improvement goals (NHC, 2021). Hospitals at all levels across the country have been encouraged to implement relevant management measures. In 2023, the national utilization rate of IV infusion among hospitalized patients was 86.24%, a decrease of 2.68% compared to 2020. The average number of IV infusion bottles/bags per bed-day among hospitalized patients nationwide was 2.87, an increase of 0.09 bottles/bags compared to 2020 (2.68 bottles/bags) (Wang, 2025). In 2023, the national medical quality and safety improvement goal underwent a strategic shift from “reducing the utilization rate of IV infusion among hospitalized patients” to “improving the standardized utilization rate of IV infusion among hospitalized patients,” emphasizing connotation management rather than merely reducing the utilization rate indicator (NHC, 2023). This implies that how to scientifically and objectively evaluate the “standardization level” of IV infusion in a hospital has become a key aspect of implementing national quality control goals. Currently, there is a lack of a unified, quantifiable, comprehensive evaluation tool in China; most studies still rely on a single utilization rate indicator for horizontal comparisons, making it difficult to reflect the balance between clinical needs and safety.

To address this critical gap, this study aims to develop a multidimensional, evidence-based evaluation framework that integrates both quantitative and qualitative indicators to assess the appropriateness of IV infusion use in general hospitals. Unlike previous studies that focused solely on utilization rates, our research innovatively employs factor analysis and cluster analysis to construct a composite scoring system that captures the complexity of infusion practices. This approach not only aligns with the national strategic shift toward “standardized utilization” but also provides a replicable and scalable tool for hospitals to identify deficiencies, guide targeted interventions, and ultimately enhance patient safety.

Therefore, based on the framework of the 2020 quality control indicators issued by the National Health Commission and the 2023 Monitoring Indicators for Inpatient Intravenous Infusion Use, this study selected nine accessible and clearly oriented IV infusion indicators (NHC, 2023; NHC, 2020). Using factor analysis and cluster analysis, we conducted a multicenter cross-sectional survey on the clinical application of IV infusion in 25 general hospitals in Shanxi Province, aiming to: (1) Develop a comprehensive evaluation method that considers both “quantity” and “quality” of IV infusion practices, moving beyond single-metric assessments; (2) Provide evidence-based references for hospitals at all levels to accurately identify their shortcomings in infusion management and formulate differentiated intervention strategies. (3) Contribute a novel, statistically robust framework to the global discourse on rational drug use, with potential applications in other healthcare systems facing similar challenges.

## 2 Methods

### 2.1 Data source

**FIGURE 1:**
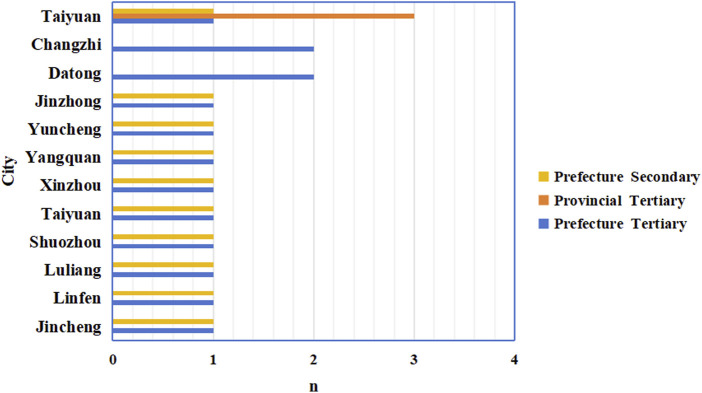
Geographic and hospital-level distributions of the 25 study hospitals.

This study focused on secondary general hospitals (SGHs) and tertiary general hospitals (TGHs) in Shanxi, China. From December 1, 2022, to January 31, 2023, 2,064 cases were extracted from 25 general hospitals (3 provincial tertiary general hospitals, 13 prefecture tertiary general hospitals, and 9 prefecture secondary general hospitals). In our previous study, the corresponding questionnaires were completed based on the case information, and the data were standardized (Duan et al., 2025). Data were retrieved from the established Shanxi Provincial Database for Antimicrobial and IV Infusion Surveys, including generic drug names, single doses, frequency, route, start and stop times of infusion orders, and calculated indicators. The study was approved by the ethics committees of all participating hospitals (Central ethics reference: (2023) YX No. 334). Detailed geographic and hospital-level distributions of the study hospitals are displayed in Figure 1. Provincial tertiary hospitals are coded S1–S3; prefecture tertiary hospitals are coded DS1–DS13; prefecture secondary hospitals are coded DE1–DE9.

### 2.2 Indicator selection and definitions

First, we classified all intravenous infusion orders according to their ATC codes. Since Traditional Chinese Medicine injections lack an ATC code, we abbreviated them as TCM. We then calculated the following indicators for each hospital: IVO: Proportion of IV infusion order entries; IVU: IV infusion utilization rate; IVB: Proportion of bed-days with IV infusion; IVN: Average number of IV infusions per bed-day (bags/bottles); IVV: Average volume of IV infusions per bed-day (mL); IVD: Average number of IV drug varieties per patient; DOT: DOT/1,000 patient-days; LOT: LOT/1,000 patient-days; DLR: DOT/LOT ratio.

Days of Therapy (DOT) for intravenous infusion is defined as the cumulative number of days a patient receives each distinct intravenous infusion product (Monnier et al., 2018). Any administration of a given product within a 24-h period counts as one DOT for that product. To describe cumulative exposure, DOT is divided by the total number of inpatient-days observed and multiplied by 1,000, yielding the number of days a specific product would be administered per 1,000 inpatient-days.

Length of Therapy (LOT) is the total number of days a patient receives any intravenous infusion, irrespective of the number of distinct products used (Tiwari and Tiwari, 2024). Higher DOT and LOT values indicate longer durations of intravenous therapy. The DOT/LOT ratio assesses the extent of combination intravenous therapy; a ratio of 1 indicates monotherapy, whereas a ratio >1 indicates combination therapy (Chiusaroli et al., 2024; Leão, 2020).

Calculations for all other indicators follow the 2020 National Quality Control Indicators for Pharmaceutical Care and the 2023 Monitoring Indicators for Inpatient Intravenous Infusion Use (NHC, 2020; NIHA, 2023).

### 2.3 Statistical analysis

All statistical analyses were performed using SPSS 27.0. The normality of continuous variables was assessed using the Shapiro-Wilk test. Non-normally distributed variables were described using the median and interquartile range [M (IQR)]. Inter-group comparisons used one-way ANOVA or non-parametric tests as appropriate. P < 0.05 was considered statistically significant; pairwise *post hoc* tests with Bonferroni correction were applied where overall differences were significant.

Principal component factor analysis: Kaiser–Meyer–Olkin (KMO) and Bartlett’s test of sphericity were applied to the nine variables to verify sampling adequacy (KMO >0.5) and variable correlation (P < 0.05). Factors with eigenvalues >1 were extracted. Varimax rotation was used to obtain the rotated factor-loading matrix; loadings ≥0.30 were retained (Liang et al., 2024). Component-score coefficient matrices were then employed to calculate factor scores for each hospital.

Cluster analysis: The derived factor scores served as clustering variables. K-means cluster analysis was conducted. Statistical description and comparison. Normality was assessed with the Shapiro–Wilk test.

## 3 Result

### 3.1 Distribution of intravenous (IV) infusion indicators

**FIGURE 2:**
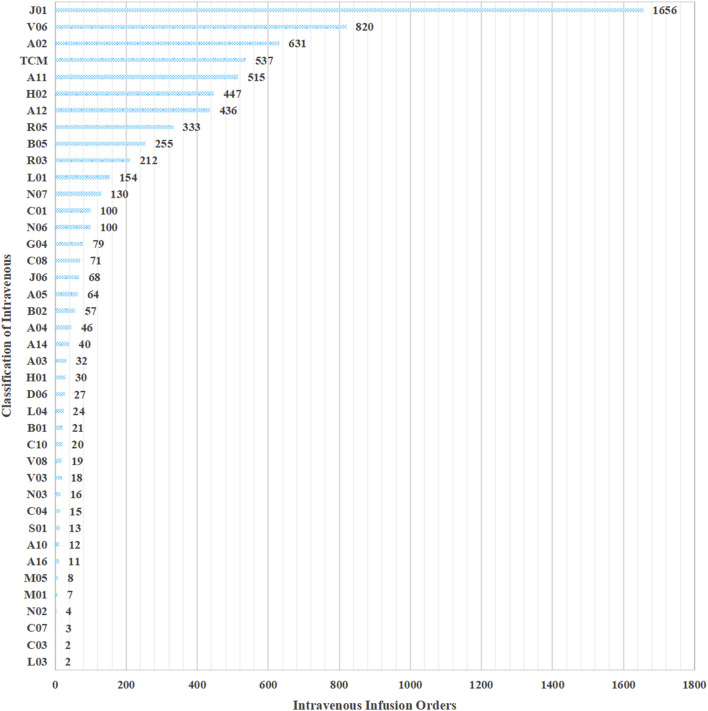
Distribution of intravenous infusion order entries by type.

When intravenous infusion orders were classified according to ATC codes, anti-infectives (J01) ranked first by a wide margin, accounting for 1,656 orders—more than twice the count of the second-ranked category, nutritional preparations (V06, 820 orders). Because traditional Chinese medicine preparations lack an official ATC designation, they were assigned to a separate category,. Acid-related disorder drugs (A02) and traditional Chinese-medicine injections (TCM, separately coded because no official ATC class exists) follow, with 631 and 537 orders, respectively. Vitamins (A11) and systemic corticosteroids (H02) complete the top six, each exceeding 400 orders. Beyond these high-volume classes, the frequencies decline steeply: only six additional ATC groups (A12, R05, B05, R03, L01, and N07) surpass 150 orders, while the remaining 30 classes contribute fewer than 100 orders each and are collectively responsible for the long right-hand tail. Overall, the pronounced concentration in the first five categories (J01, V06, A02, TCM, and A11) illustrates that IV therapy in the audited hospitals is largely driven by anti-infective prophylaxis/treatment, nutritional support, acid-suppression, and TCM protocols, highlighting priority areas for future stewardship interventions. The detailed ATC breakdown for each class is provided in Figure 2.

Across different hospitals, the ranges of IV infusion indicators are as follows: proportion of IV infusion orders, 31.68%–61.00%; IV infusion utilization rate, 66%–100%; proportion of bed-days with IV infusion, 62.14%–100%; average number of IV bags/bottles per bed-day, 1.72-4.52; average volume per bed-day, 278.93–789.36 mL; average number of drug varieties per patient, 2.37-5.05; DOT/1000 patient-days, 1193.00 -3083.27; LOT/1000 patient-days, 621.00 -1000.00; DOT/LOT ratio, 1.70 -3.47. Provincial centres (S1–S3) showed the lowest proportion of IV orders (IVO 31.7%–44.0%) and the smallest average number of bags per bed-day (IVN 2.52–4.52), whereas secondary hospitals (DE1–DE9) consistently achieved 100% IV utilisation (IVU) and the highest IVO (up to 61% in DE7). The distribution of intravenous infusion indicators in each hospital is shown in Table 1.

**TABLE 1:** Distribution of intravenous infusion indexes in each hospital.

Hospital	Intravenous infusion indicators								
	IVO (%)	IVU (%)	IVB (%)	IVN	IVV	IVD	DOT	LOT	DLR
S1	31.68	86.00	74.05	2.87	432.89	3.75	1842.98	740.55	2.49
S2	44.01	98.00	95.02	4.52	786.36	5.05	3083.27	950.15	3.25
S3	34.91	84.69	73.29	2.52	377.56	3.69	1954.22	732.86	2.67
DS1	50.83	97.00	100.00	3.21	528.02	3.35	2039.75	1000.00	2.04
DS2	46.05	98.00	86.89	3.38	649.83	4.51	2438.52	868.95	2.81
DS3	37.14	96.00	84.04	2.59	488.06	2.86	1426.17	840.42	1.70
DS4	33.37	89.00	86.72	2.88	629.93	2.83	1656.74	867.23	1.91
DS5	41.44	91.00	85.47	2.92	449.64	3.40	2218.31	854.71	2.60
DS6	42.94	66.00	69.21	3.10	555.20	3.02	2401.07	692.10	3.47
DS7	35.76	93.94	90.62	4.19	528.41	4.47	2912.01	906.21	3.21
DS8	50.00	83.84	65.42	2.70	278.93	3.16	1810.42	654.17	2.77
DS9	37.98	85.32	62.15	1.72	513.38	2.50	1193.46	621.48	1.92
DS10	41.63	94.00	79.03	3.07	620.12	4.00	2207.35	790.33	2.79
DS11	39.85	96.00	83.24	2.70	718.51	3.53	2026.00	832.38	2.43
DS12	43.83	89.00	79.72	2.43	602.68	2.37	1497.22	797.22	1.88
DS13	33.06	80.00	85.28	2.77	441.83	2.79	2081.80	852.76	2.44
DE1	41.13	100.00	100.00	3.05	372.24	3.84	2259.52	1000.00	2.26
DE2	43.03	96.00	98.36	3.81	294.93	3.54	2616.39	983.61	2.66
DE3	33.55	81.82	62.14	1.74	549.62	2.78	1431.55	621.44	2.30
DE4	47.24	94.44	97.03	3.84	483.69	3.69	2883.72	970.31	2.97
DE5	36.68	84.00	83.10	3.23	547.43	3.72	2565.61	831.01	3.09
DE6	35.52	96.00	96.65	2.49	535.60	2.74	1761.99	966.47	1.82
DE7	61.00	100.00	90.89	3.27	364.38	3.58	2432.91	908.86	2.68
DE8	47.10	100.00	98.75	3.51	420.97	4.74	2908.20	987.47	2.95
DE9	47.89	94.00	100.00	3.55	640.60	3.60	2960.61	1000.00	2.96
$\bar{X}\pm s$	41.50 ± 6.92	90.96 ± 8.04	85.08 ± 12.08	3.04 ± 0.66	512.43 ± 125.58	3.50 ± 0.69	2184.37 ± 535.65	850.72 ± 120.83	2.56 ± 0.49

Provincial-level third-level medical institutions are S1∼S3, prefecture-level third-level medical institutions are DS1∼DS13, and prefecture-level second-level medical institutions are DE1∼DE9; IVO: Proportion of IV infusion order entries; IVU: IV infusion utilization rate; IVB: Proportion of bed-days with IV infusion; IVN: Average number of IV infusions per bed-day (bags/bottles); IVV: Average volume of IV infusions per bed-day (mL); IVD: Average number of IV drug varieties per patient; DOT: DOT/1,000 patient-days; LOT: LOT/1,000 patient-days; DLR: DOT/LOT ratio.

### 3.2 Results of principal component factor analysis

In the adaptability test of the principal component factor analysis, the Kaiser-Meyer-Olkin (KMO) value for the nine intravenous infusion indicators was 0.706. The Bartlett’s test of sphericity yielded an approximate chi-square value of 495.497, with 36 degrees of freedom and p < 0.01, indicating that the assumption of independence among the nine variables was rejected. This suggests a strong correlation among the variables, making them suitable for principal component analysis.

Using principal component analysis, the component matrix and eigenvalues were calculated. Two principal components with eigenvalues greater than 1 were retained. With CF1 explained 61.541% of the total variance (rotated eigenvalue = 3.970), CF2 explained 20.372% (rotated eigenvalue = 3.402), collectively accounting for 81.913% of the cumulative variance (Table 2). This indicates that the first two principal components account for more than four-fifths of the information contained in the nine intravenous infusion indicators.

**TABLE 2:** Variances, contribution rate, and cumulative contribution rate of the correlation coefficient.

Component	Initial eigenvalues			Rotation sums of squared loading		
	Eigenvalue	Variance contribution rate (%)	Cumulative variance contribution rate (%)	Eigenvalue	Variance contribution rate (%)	Cumulative variance contribution rate (%)
CF1	5.539	61.541	61.541	3.970	44.110	44.110
CF2	1.834	20.372	81.913	3.402	37.803	81.913

CF, common factor.

Next, the principal component matrix was rotated using the varimax rotation method to obtain the factor loading matrix. A threshold of factor loading >0.30 was applied to identify characteristic variable factor groups, as shown in Table 3. Rotated factor pattern and score coefficients. CF1 (“intensity”) is dominated by volume-related variables (IVN λ = 0.83, IVV λ = 0.80, DOT λ = 0.90), whereas CF2 (“penetration”) is anchored by coverage metrics (IVU λ = 0.90, IVB λ = 0.92). The score-coefficient signs reveal that higher raw values on IVN, IVV, and DLR increase CF1, while higher IVU and IVB increase CF2. The data of 9 intravenous infusion indicators of 25 general hospitals in Table 1 were standardized. According to the factor score coefficient matrix (Table 3), the calculation models of the two common factors were obtained (ZXn represents the standardized data of Xn):

$$F1=0.032*{\text{ZX}}_{\text{IVU}}-0.150*{\text{ZX}}_{\text{IVN}}-0.073*{\text{ZX}}_{\text{IVV}}+0.193*{\text{ZX}}_{\text{DOT}}+0.180*{\text{ZX}}_{\text{LOT}}+0.212*{\text{ZX}}_{\text{DLR}}+0.237*{\text{ZX}}_{\text{IVO}}-0.073*{\text{ZX}}_{\text{IVD}}+0.361*{\text{ZX}}_{\text{IVB}};$$ (1)

$$F2=0.099*{\text{ZX}}_{\text{IVU}}+0.345*{\text{ZX}}_{\text{IVN}}+0.309*{\text{ZX}}_{\text{IVV}}+0.036*{\text{ZX}}_{\text{DOT}}+0.047*{\text{ZX}}_{\text{LOT}}-0.020*{\text{ZX}}_{\text{DLR}}-0.019*{\text{ZX}}_{\text{IVO}}+0.309*{\text{ZX}}_{\text{IVD}}-0.258*{\text{ZX}}_{\text{IVB}}$$ (2)

F=F1*44.11/81.91+F2*37.803/81.91 (3)

**TABLE 3:** Rotated factor loading matrix and coefficient matrix.

Indicators	Rotated factor loading matrix		Coefficient matrix	
	CF1	CF2	CF1	CF2
IVO	0.307	**0.395**	0.032	0.099
IVU	0.037	**0.900**	−0.150	0.345
IVB	0.276	**0.918**	−0.073	0.309
IVN	**0.831**	0.475	0.193	0.036
IVV	**0.801**	0.489	0.180	0.047
IVD	**0.805**	0.321	0.212	−0.020
DOT	**0.904**	0.368	0.237	−0.019
LOT	0.276	**0.918**	−0.073	0.309
DLR	**0.962**	−0.216	0.361	−0.258

IVO: Proportion of IV infusion order entries; IVU: IV infusion utilization rate; IVB: Proportion of bed-days with IV infusion; IVN: Average number of IV infusions per bed-day (bags/bottles); IVV: Average volume of IV infusions per bed-day (mL); IVD: Average number of IV drug varieties per patient; DOT: DOT/1,000 patient-days; LOT: LOT/1,000 patient-days; DLR: DOT/LOT ratio. Bold values indicate the dominant component (CF1 or CF2) on which the respective indicator is judged to load.

In Equation 1, the positive coefficients 0.361 (ZX_IVB_) and 0.237 (ZX_IVO_>) indicate that higher “bed-day coverage” and “order proportion” elevate F1, whereas the negative coefficients −0.150 (ZX_IVN_) and −0.073 (ZX_IVV_) show that F1 increases when “bags per bed-day” or “volume” decrease. Hence, F1 is labelled the “intensity” factor, and lower scores signal better intensity control. In Equation 2, the coefficients of ZX_IVN_, ZX_IVV,_ and ZX_IVD_ are all positive and >0.30, meaning that more bags, larger volume, or more drug varieties raise F2 simultaneously. In contrast, the coefficient −0.258 for ZX_IVB_ implies that excessive bed-day coverage actually lowers F2, reflecting that F2 emphasises “penetration depth” rather than sheer breadth; it is therefore defined as the “penetration” factor. Equation 3 weights the two factors by their variance contributions (CF1 44.11%, CF2 37.80%) to synthesise a composite score F on a 0%–100% scale. A lower F indicates superior performance on both “low intensity + low penetration” dimensions and can be fed directly into subsequent K-means clustering and performance grading.

Since the nine IV infusion-related indicators are oriented toward gradual decline, the composite factor score F was ranked from lowest to highest to assess the ranking of hospitals in terms of the rational use of IV infusion for inpatients (Table 4). The top three hospitals for rational IV infusion use were DS9, DE3, and DS12, whereas the bottom three were DS7, DE8, and S2, suggesting substantial room for improvement in IV infusion practices.

**TABLE 4:** Factor analysis composite scores of 25 hospitals.

Hospital code	F1	F2	F	Ranking
DS9	−1.550	−1.218	−1.386	1
DE3	−1.051	−1.653	−1.314	2
DS12	−1.432	0.003	−0.770	3
DS8	0.092	−1.322	−0.549	4
S3	−0.082	−1.099	−0.542	5
DS3	−1.436	0.567	−0.517	6
DS4	−1.078	0.233	−0.475	7
S1	−0.113	−0.912	−0.474	8
DS13	−0.400	−0.541	−0.461	9
DE6	−1.591	1.068	−0.373	10
DS6	1.302	−2.333	−0.355	11
DS11	−0.305	0.162	−0.091	12
DS5	−0.007	0.003	−0.002	13
DS10	0.314	−0.322	0.023	14
DE5	0.941	−0.724	0.179	15
DS1	−0.675	1.457	0.296	16
DE1	−0.267	1.338	0.462	17
DE7	0.191	0.918	0.519	18
DS2	0.766	0.362	0.576	19
DE2	0.313	0.909	0.580	20
DE4	0.873	0.681	0.778	21
DE9	0.792	0.821	0.798	22
DS7	1.578	0.073	0.883	23
DE8	0.863	0.940	0.890	24
S2	1.963	0.590	1.324	25

### 3.3 K-means clustering results

**FIGURE 3:**
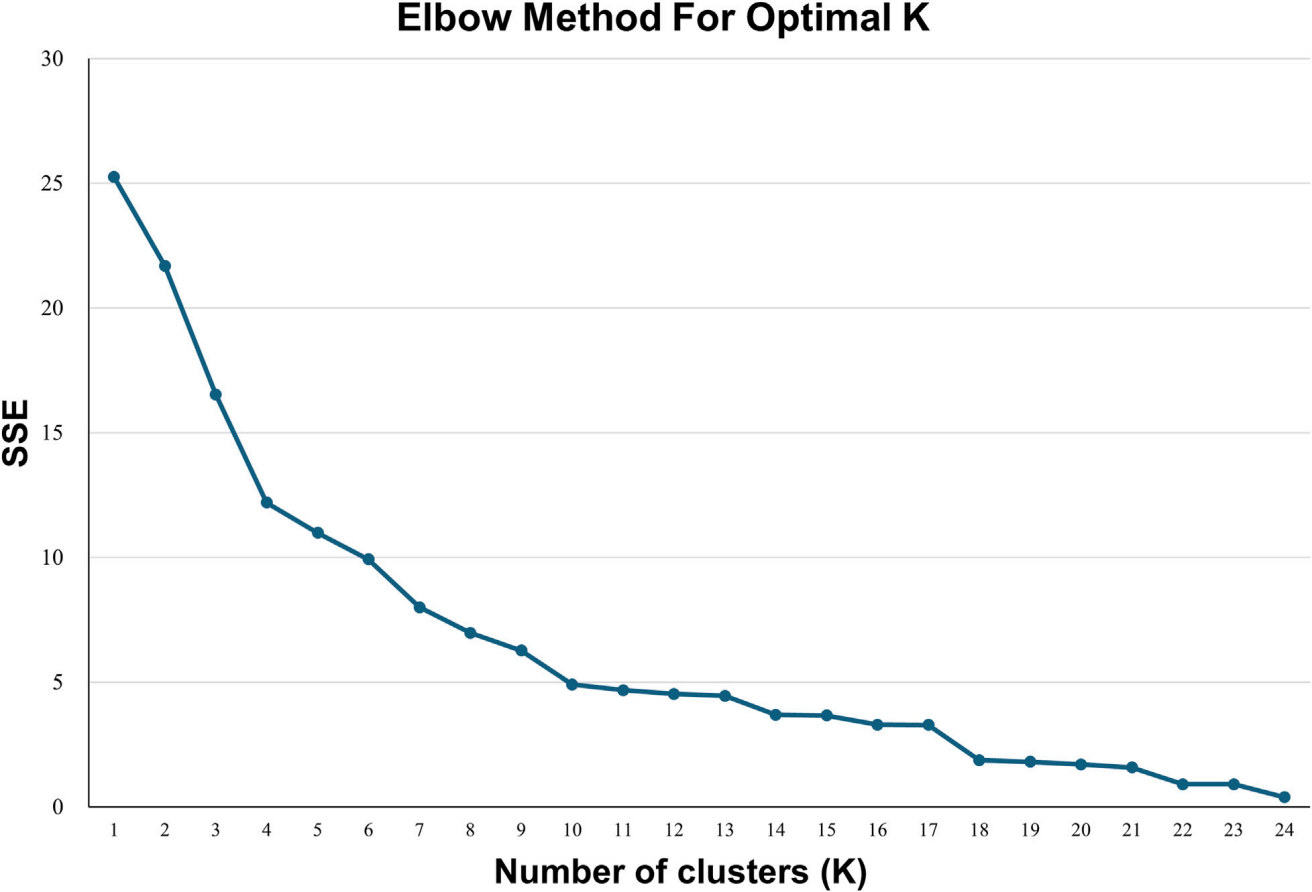
Elbow chart.

After reducing the dimension of the data using principal component factor analysis, the scores of the two common factors of all hospitals were used as new clustering variables for K-means clustering analysis. The elbow method was used to draw the elbow plot. As can be seen from Figure 3, when K = 4, the degree of distortion changes the most. After exceeding 4, the change in the degree of distortion significantly decreases. Therefore, the number of categories can be set to 4.

**FIGURE 4:**
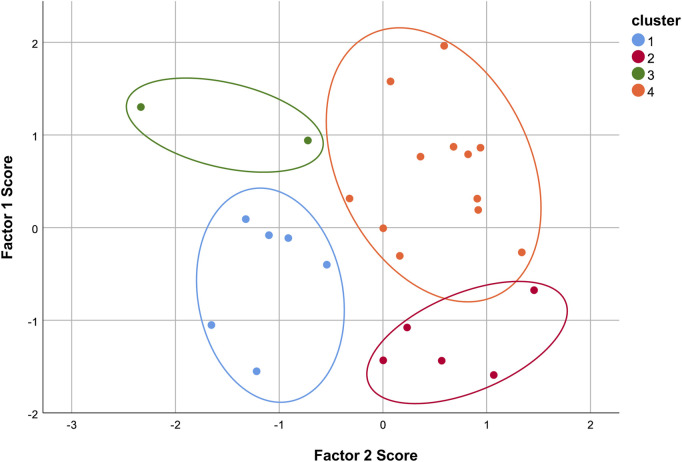
K-Means cluster analysis results.

As shown in Figure 4, the 25 hospitals can be classified into 4 categories through K-Means clustering. Six hospitals are classified into the first category, five into the second, two into the third, and twelve into the fourth. The distribution of the comprehensive evaluation of each hospital through factor analysis in the cluster analysis is presented in Table 5. The F values of the four types of hospitals are −0.788 ± 0.438, 0.368 ± 0.399, −0.088 ± 0.378, and 0.561 ± 0.420, respectively.

**TABLE 5:** The distribution of factor analysis comprehensive evaluation in cluster analysis.

Hospital code	Score	Cluster	Ranking	Cluster	$\bar{X}\pm s$
DS9	−1.386	1	1	Excellent	−0.788 ± 0.438
DE3	−1.314	1	2	Excellent	
DS8	−0.549	1	4	Excellent	
S3	−0.541	1	5	Excellent	
S1	−0.473	1	8	Excellent	
DS13	−0.461	1	9	Excellent	
DS12	−0.77	2	3	Good	−0.368 ± 0.399
DS3	−0.517	2	6	Good	
DS4	−0.475	2	7	Good	
DE6	−0.373	2	10	Good	
DS1	0.296	2	16	Good	
DS6	−0.355	3	11	Middle	−0.088 ± 0.378
DE5	0.179	3	15	Middle	
DS11	−0.091	4	12	Inferior	0.561 ± 0.420
DS5	−0.002	4	13	Inferior	
DS10	0.023	4	14	Inferior	
DE1	0.462	4	17	Inferior	
DE7	0.519	4	18	Inferior	
DS2	0.576	4	19	Inferior	
DE2	0.58	4	20	Inferior	
DE4	0.778	4	21	Inferior	
DE9	0.798	4	22	Inferior	
DS7	0.883	4	23	Inferior	
DE8	0.8902	4	24	Inferior	
S2	1.3242	4	25	Inferior	

Table 6 presents the results of the variance analysis and non-parametric test for the four types of hospitals. The results show that, except for the proportion of the “intravenous infusion prescription items” which showed no statistically significant difference among the various hospitals (P = 0.131), the other eight indicators all showed statistically significant differences (P < 0.01). The Excellent group (n = 6) used on average 374.52 mL IV fluid per bed-day—almost 40% less than the Inferior group (603.78 mL, p < 0.01). Despite lower volume, the Excellent group achieved comparable clinical coverage (IVB 70% vs. 92%, p < 0.01), suggesting that judicious patient selection and early oral switch can maintain therapeutic efficacy while reducing exposure. Based on the ranking and scoring of the indicators’ distribution levels among the various hospitals, a score of 4 points to 1 point was assigned from low to high. The results indicated that the first type of hospital scored 32 points and was named the excellent group; the second type of hospital had an overall score of 26 points and was named the good group; the third type of hospital had an overall score of 22 points and was named the average group; the fourth type of hospital had an overall score of 10 points and was named the poor group.

**TABLE 6:** The overall situation of 9 evaluation indicators in each cluster group.

Indicators	Total	Excellent(n = 6)	Good (n = 5)	Middle (n = 2)	Inferior (n = 12)	*P*
IVO	41.50 ± 6.92	36.86 ± 6.78	40.14 ± 7.14	39.81 ± 4.43	44.68 ± 6.25	0.131
IVU	94.00[85.01,96.50]	84.27[81.37,85.49]^a^ ^(d)^	96.00[89.00,96.50]^b^	75.00[66.00,-]^c(d)^	96.00[94.00,99.50]^d(a,c)^	<0.01
IVB	85.08 ± 12.08	70.39 ± 8.99^a(b,d)^	89.43 ± 8.58^b(a)^	76.16 ± 9.82^c^	92.11 ± 7.17^d(a)^	<0.01
IVN	3.04 ± 0.66	2.39 ± 0.52^a^ ^(d)^	2.72 ± 0.32^b(d)^	3.17 ± 0.09^c^	3.48 ± 0.54^d(a,b)^	<0.01
IVV	512.43 ± 125.58	374.52 ± 71.52^a^ ^(d)^	440.47 ± 71.52^b(d)^	558.03 ± 63.14^c^	603.78 ± 87.46^d(a,b)^	<0.01
IVD	3.50 ± 0.69	3.11 ± 0.52^a^ ^(d)^	2.83 ± 0.35^b(d)^	3.37 ± 0.49^c^	4.00 ± 0.55^d(a,b)^	<0.01
DOT/1000pd	2184.37 ± 535.65	1718.96 ± 3^a^7.65^a^ ^(d)^	1676.33 ± 242.14^b(c,d)^	2483.54 ± 116.62^c(b)^	2578.89 ± 360.68^d(a,b)^	<0.01
LOT/1000pd	850.72 ± 120.83	703.83 ± 90.21^a(b,d)^	894.00 ± 85.84^b(a)^	761.50 ± 98.29^c^	921.00 ± 71.71^d(a)^	<0.01
DLR	2.56 ± 0.49	2.43 ± 0.30^a(b,c)^	1.87 ± 0.12^b(a,c,d)^	3.28 ± 0.27^c(a,b)^	2.80 ± 0.29^d(b)^	<0.01

a = Excellent; b = Good; c = Middle; d = Inferior.

^a∼d^ represents the statistical difference of pair-to-pair comparison between hospitals (Bonferroni p < 0.05).

## 4 Discussion

Cluster analysis—an unsupervised machine-learning technique that recovers latent subgroup structures without *a priori* labels—has become a cornerstone of healthcare and pharmaceutical decision-making. Sanyaolu (2025) demonstrated that, by aggregating unlabelled data according to intrinsic similarity, cluster algorithms can reveal previously obscured patient or product phenotypes that elude conventional risk-stratification tools. Valderas et al. (2018) applied K-means to 520,000 primary-care records and found six multimorbidity-drug–drug patterns; the cluster combining metabolic disorders, cardiovascular disease and NSAIDs had the highest hospitalisation rate, guiding pathway redesign. Baldacci et al. (2024) used K-means to split 1,200 hospital drugs into high, medium, and low demand-volatility groups, set stock levels accordingly, and cut stock-outs by 21% while boosting turnover by 16%. Gonzalez and Gonzalez (2015) classified 14,000 antibiotic prescriptions into high, moderate, and low risk with Two-Step clustering; reviewing the high-risk group prospectively reduced prescribing errors by 34%. Burt et al. (2023) embedded an alternating logistic-regression cluster model in a worker cohort and identified four rotator-cuff-disease clusters; high-repetition lifting with short rest carried a 2.3-fold higher incidence, providing early warning signals. Together, these studies show clustering converts complex health data into actionable groups, supporting evidence-based regulation, resource optimisation, and safer care.

Our study explored the feasibility of using multidimensional statistical methods to evaluate whether hospitals’ intravenous infusions are appropriate. The 25 hospitals were classified into four performance tiers—excellent, good, fair, and poor—with 48% falling into the “poor” tier, indicating substantial room for improving infusion standardization across the province. Notably, top performance on any single indicator did not guarantee a top composite rank: DS9 Hospital, for example, ranked first overall despite never placing first on any of the nine individual indicators, owing to balanced scores across all dimensions. Conversely, S2 Hospital—whose utilization rate was not the highest—finished last because of multidimensional imbalance. This finding aligns closely with the 2023 national goal of “enhancing appropriate use rates” rather than “simply reducing use rates,” underscoring the need to shift evaluation from single indicators to composite metrics.

Overseas studies have repeatedly documented the harms of excessive intravenous therapy. A systematic review by Sutherland et al. reported a weighted incidence of 101 medication errors per 1,000 IV administrations among British in-patients (Clarke et al., 2018), while an Australian multicentre study found that patients with a DOT/LOT ratio >2 had a higher risk of drug-related adverse events (Santos, 2020). In the present study, hospitals rated as “poor” had a mean DOT/LOT of 2.80, suggesting that excessive combination therapy is a major source of safety hazards. Domestically, Zuo et al. (NMPA, 2024) reported a 2020 national average of 2.69 bottles per bed-day (Zuo Wei and Gao, 2023); the Shanxi sample in this study averaged 3.04 bottles, exceeding the national figure and consistent with previous single-centre reports from East and South China (Sun et al., 2019), indicating that northern secondary-and-above hospitals generally infuse at high intensity. We further used factor analysis to distill the nine indicators into an “infusion-intensity factor” and a “penetration factor”, retaining 81.9% of the original variance while eliminating multicollinearity—an approach that aligns with WHO guidance on composite-indicator construction (Shrestha, 2021).

This cross-sectional study drew data only from December 2022 to January 2023, so findings may have been distorted by post-COVID resource shortages; moreover, non-surgical wards alone were analysed, peri-operative infusion appropriateness was not assessed, and the absence of indication and outcome data precludes causal inferences. Future work should prolong the observation window, enlarge the sample to include surgical, oncological, and paediatric patients, link real-world data to prospectively quantify the causal pathway between infusion exposure and ADRs, length of stay, or readmission, and develop an AI-driven dynamic early-warning system for real-time risk mitigation. Aligned with the latest national quality-control targets, we provide an evidence-based, readily implementable evaluation model that offers a practical roadmap for refining intravenous-infusion management in Shanxi and similar settings nationwide.

## 5 Conclusion

This study presents a multidimensional, evidence-based framework for evaluating the appropriateness of inpatient intravenous (IV) infusion use across hospitals. By integrating nine key indicators through factor and cluster analyses, we developed a composite scoring system that reflects both the intensity and penetration of IV therapy. The model successfully classified 25 general hospitals in Shanxi Province into four performance tiers, revealing that nearly half still fall into the “poor” category, with excessive combination therapy and high utilization rates being major contributors. These findings underscore the importance of shifting from single-indicator assessments to comprehensive, balanced evaluations in line with national quality improvement goals. The proposed tool offers a practical, scalable approach for hospitals nationwide to identify gaps, guide targeted interventions, and ultimately enhance the safety and rationality of IV infusion practices.

## Data Availability

The raw data supporting the conclusions of this article will be made available by the authors, without undue reservation.
